# Keshan Disease: A Potentially Fatal Endemic Cardiomyopathy in Remote Mountains of China

**DOI:** 10.3389/fped.2021.576916

**Published:** 2021-03-09

**Authors:** Ying Shi, Wei Yang, Xianwen Tang, Quanhao Yan, Xiaojing Cai, Fenfang Wu

**Affiliations:** ^1^Department of Central Laboratory, Shenzhen Hospital, Beijing University of Chinese Medicine, Shenzhen, China; ^2^Department of Physical Examination, Shenzhen Hospital, Beijing University of Chinese Medicine, Shenzhen, China; ^3^Department of Cardiovascular Medicine, Shenzhen Hospital, Beijing University of Chinese Medicine, Shenzhen, China

**Keywords:** Keshan disease, cardiomyopathy, endemic, etiological, environment

## Abstract

Keshan disease (KD) as an endemic, highly lethal cardiomyopathy, first reported in northeast China's Keshan County in 1935. The clinical manifestations of patients with KD include primarily congestive heart failure, acute heart failure, and cardiac arrhythmia. Even though some possible etiologies, such as viral infection, fungal infection, microelement deficiency, and malnutrition, have been reported, the exact causes of KD remain poorly known. The endemic areas where KD is found are remote and rural, and many are poor and mountainous places where people are the most socioeconomically disadvantaged in terms of housing, income, education, transportation, and utilization of health services. To date, KD is a huge burden to and severely restricts the economic development of the local residents and health systems of the endemic areas. Although efforts have been made by the government to control, treat, and interrupt disease transmission, the cure for or complete eradication of KD still requires global attention. For this reason, in this review, we systematically describe the etiological hypothesis, clinical manifestations, incidence characteristics, and treatment of KD, to facilitate the better understanding of and draw more attention to this non-representative cardiovascular disease, with the aim of accelerating its elimination.

## Introduction

Keshan disease (KD) is an endemic cardiomyopathy with high fatality rates, first reported in Keshan County in China in 1935 (1). Nationwide, KD has been reported in 2,953 towns in 327 counties in 16 provinces (municipalities and autonomous regions) from northeast to southwest, a band area. These KD-endemic areas contain approximately 60.487 million people (2). The average annual incidence was 10/100,000 population (3). In 1960, the worst incidence of KD in the Chuxiong region of Yunnan Province exceeded 100/100,000, and the mortality rate exceeded 98%.

The clinical manifestations of KD are acute or chronic episodes of heart disease characterized by cardiogenic shock, congestive heart failure, and arrhythmia, along with cardiomegaly (4). Based on the onset, cardiac function, clinical manifestations, or pathological results, the etiology of KD is defined as follows, divided into four types: acute KD, subacute KD, chronic KD, and latent KD (2). For acute KD, the onset is sudden, manifesting as acute heart function, cardiac insufficiency such as pulmonary edema, severe arrhythmia, and cardiogenic shock (5). Electrocardiogram (ECG) commonly reveals ST–T changes. In subacute cases, the onset is slower than in acute patients, and most cases show a “galloping” heart rhythm and facial edema (6). In recent years, chronic and latent KD have been the most two prevalent types reported. In chronic KD, the onset is slow. The patient presents with chronic heart failure, ventricular dilation, myocardial fibrosis, and a thinning heart wall. In latent KD, the episode is disguised, and the patient's cardiac function is fairly good [New York Heart Association (NYHA) class I]. Ventricular contractions and changes in the right bundle-branch block or ST–T are common. However, the etiology of KD is unknown (7).

In the last few decades, numerous investigators have explored the causes of KD, and the main etiology is believed to be selenium (Se) deficiency. This is primarily because KD usually occurs in a specific region of China and has affected individuals reporting similar Se deficiency conditions (8). Se is a trace mineral that plays a crucial role in protecting the body against oxidants, serving as an essential component of several antioxidant enzymes such as glutathione peroxidase (GPx) and glutathione reductase. An Se deficiency has also been known to contribute to coxsackievirus B3 (CVB3)–induced myocarditis in acute and subacute phases of infection. Thus, KD may have a dual etiology, with both CVB3 infection and Se deficiency being responsible for KD of the heart (9, 10). Recent studies have also indicated that CYP1A1 and CYP2C19 are highly expressed (ratios ≥2.0) in patients with KD (11). These genes belong to the cytochrome P450 isoforms, and their metabolites are biologically active and critical for the maintenance of essential bodily functions (12, 13). KD is widely considered a multifactorial environment–gene interaction complex disease. Since an outbreak of KD in 1935, resulting in speculation about and efforts to determine the etiology of the disease, KD still haunts the health of poor farmers in historically serious endemic areas.

KD is regarded as the cardiovascular disease most responsible for the high morbidity and mortality rates in China (14). All KD-endemic areas are rural, where people are the most socioeconomically disadvantaged. Patients with KD are usually among the poorest. Although KD has appeared in China for nearly a century, it has received less attention, especially by Western medical scientists, and only a few articles on the pathology of KD have been published in English. Herein, we describe the etiological hypothesis, clinical manifestations, incidence characteristics, and treatment of KD, to facilitate the understanding of and draw more attention to the eradication of this non-representative and challenging cardiovascular disease.

## Incidence Characteristics

**Figure 1:**
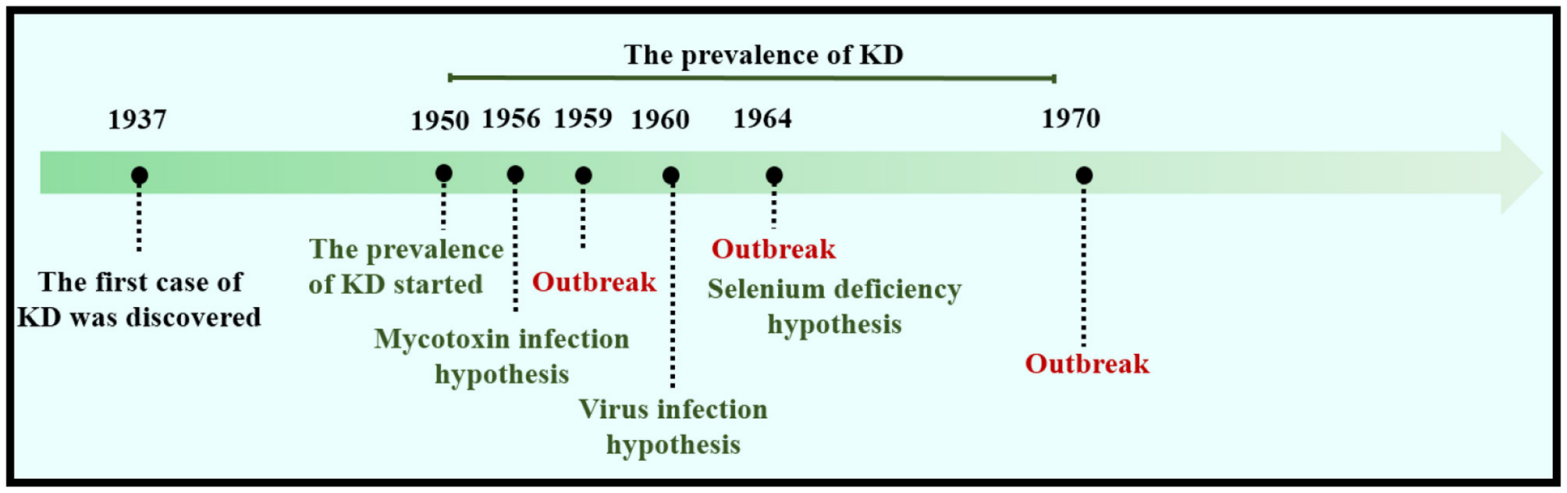
A brief history of KD.

KD was first identified in Keshan County, Heilongjiang Province, China, during the winter of 1935 (Figure 1). KD was prevalent from 1950 to 1970, with three major outbreaks, in 1959, 1964, and 1970. In 1956, Guo et al. tested the bacterial content of *Fusarium* beads in the grains in both endemic and non-endemic areas. The content of bacteriocin in the endemic areas was found to be much higher, and the fungal infection theory was proposed (15). Four years later, the coxsackievirus was found through enterovirus isolation and serum antibody tests of patients with KD, and the virus infection hypothesis prevailed. Until 1964, white muscle disease caused by Se deficiency was found in animals in areas where KD was reported, with the main pathological changes being similar to those of KD (16). Therefore, it was hypothesized that the myocardial lesions of KD are associated with Se deficiency.

It is known that KD has regional distribution, with population and seasonal fluctuations of incidence.

## Regional Distribution of KD

**Figure 2:**
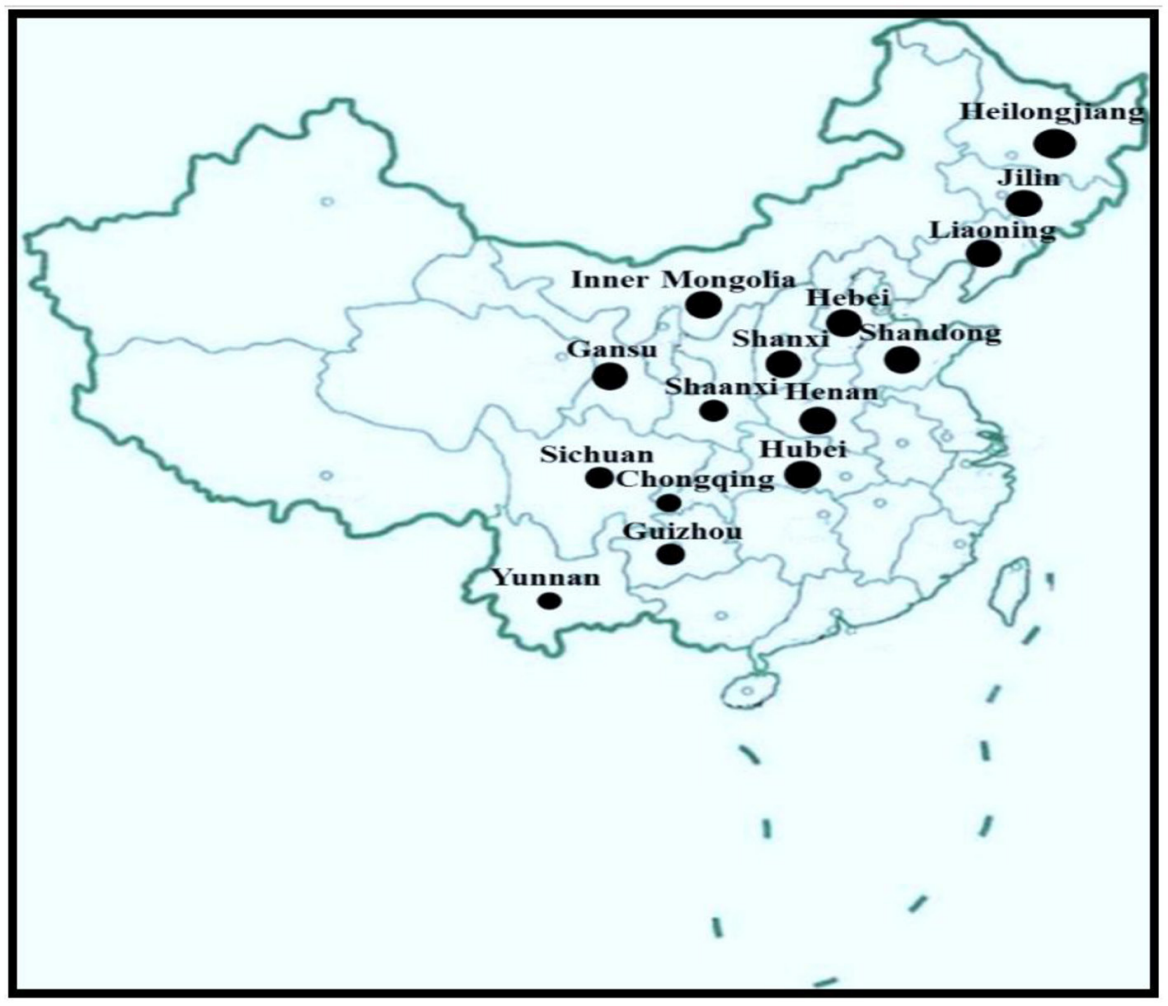
The main endemic areas of KD in China (highlighted in black).

KD has been reported in 2,953 townships in 327 counties in 15 provinces from the northeast to the southwest, only in certain areas in China, a belt zone (Figure 2). The KD-endemic areas are all remote rural mountain areas where people are the most socioeconomically disadvantaged (2). Interestingly, in the KD-endemic provinces, only a few counties and towns are KD-endemic (4, 16). According to the KD Endemic Area Definition and Classification, KD-endemic areas are often adjacent to Se-rich regions, and the affected areas within the endemic zones present as small foci (16). KD-endemic and non-endemic areas are usually separated by a hill or a river. Amid KD-endemic areas there are likely to be small non-endemic areas, called “safety islands.”

In the non-endemic areas, staple foods were mixed, suggesting that people who had been eating a “single type of food” all year round, harvested in an endemic area with its particular water-soil conditions, were liable to suffer from KD (17, 18). The soil erosion in endemic areas is serious, leading to lower levels of trace elements, minerals, vitamins, and amino acids essential for good cardiac metabolism (19). Endemic foci can always be explained by Se distribution. The survey found that the Se content of local residents' food samples was lower in endemic areas than in nearby non-endemic areas (20, 21).

## Seasonal Prevalence of KD

The incidence of KD is seasonal, with acute KD showing peaks in winter or spring in northeast rural areas and in summer in southwest rural areas in China (22). Chronic and subacute KD shows year-round prevalence. Therefore, some experts have speculated that temperature may accelerate the onset of this disease (23). The 2008 cold spell of South China was widely considered to be the most extreme of the past five decades. In an investigation reported in Guangdong, the southern province of China, three cities were selected for a study of the impact of the cold spell on cardiovascular incidents from January 2006 through December 2009. This study showed 66.2, 66.5, and 39.7% more deaths than the average for the corresponding days of the three preceding years in the three cities, indicating that temperature challenges directly affect cardiovascular health. Similarly, heat waves also play a key role in the occurrence of cardiovascular diseases (24). A survey in Sydney, Australia, showed that there was a statistically significant increase in the ratios of hospital admissions for cardiovascular reasons on hot days (25). However, doctors at Jilin Medical College reported that deaths occurring from acute KD and newly formed pathologic changes in the heart are found all year round. This indicates that the onset of KD is not dependent only on seasonal changes (26).

## Prevalent Population

Most patients with KD are among the poorest of peasants (27). Ninety-nine percent live on grains from their own fields, and 80% of these are young females of child-bearing age and infants after lactation. In the southwestern area, child-bearing females are the most susceptible. According to recent reports, the KD incidence rate was also higher in females (2.20%) than in males (1.98%) (6, 28). The higher KD incidence rates in women might be caused by their lower immunity compared with that of men (29). However, others living in the same endemic areas, such as foresters, coal miners, and railway workers, who consume commercial agricultural products rather than food produced by local peasants, did not have KD (30). Thus, KD leads to a vicious cycle of poverty and illness in remote mountain regions. Today, KD still afflicts poor farmers in remote mountainous endemic areas, causing great damage to their health and economy.

## Etiology of KD

**Figure 3:**
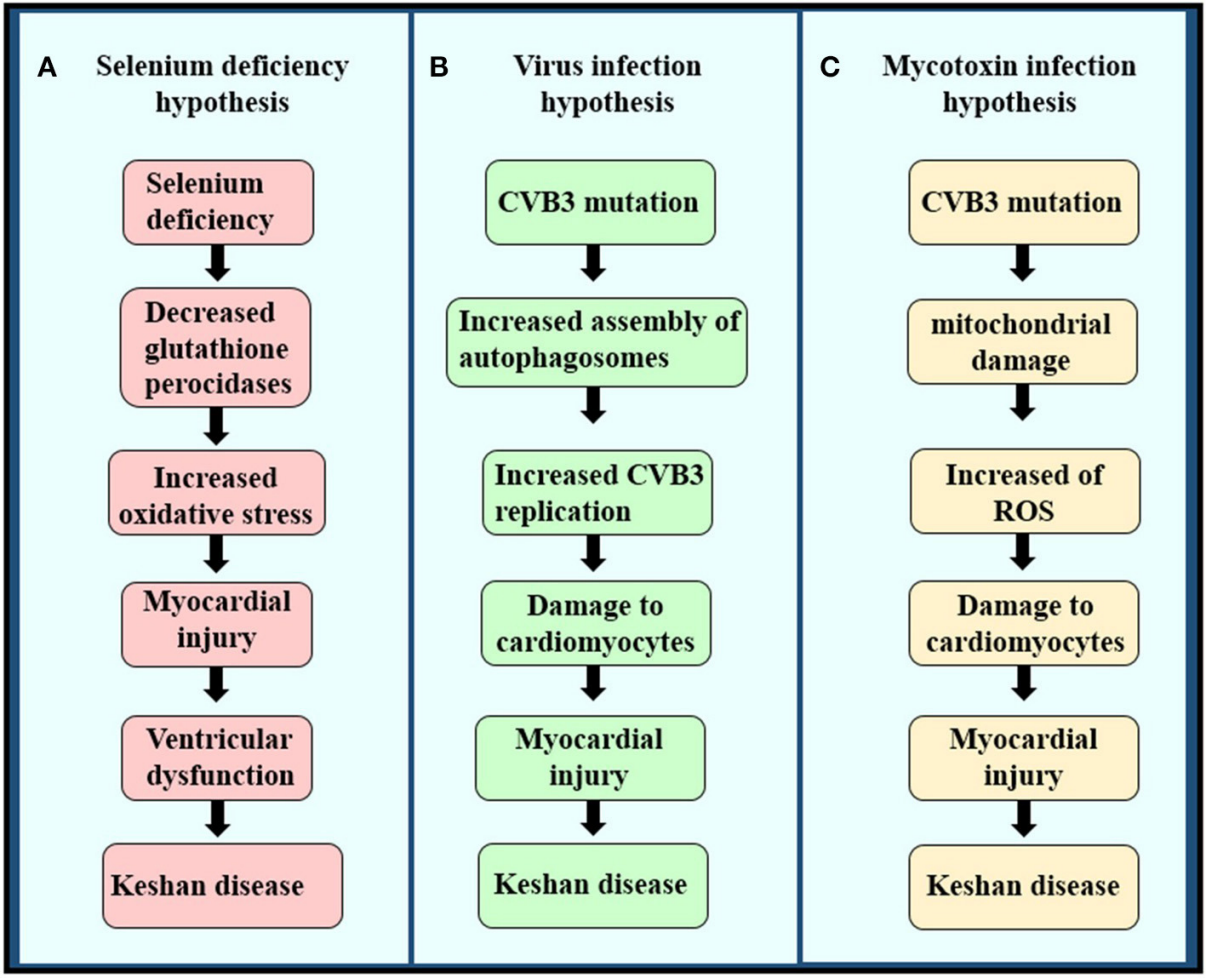
The pathogenesis of the three etiologies of KD. **(A)** Selenium deficiency hypothesis, **(B)** virus infection hypothesis, and **(C)** mycotoxin infection hypothesis.

Although KD has been reported in China for nearly a century, its cause has not been clear until now. Multiple etiological hypotheses have been proposed, including intoxication with mycotoxins or environmental toxins (31, 32), viral infection, and trace element deficiency caused by a monotonous diet lacking minerals or vitamins (32), such as magnesium, iron, or thiamin (Figures 3A–C) (18). Among the many underlying causes of KD, the hypothesis of Se deficiency is considered the most convincing (33).

## Se Deficiency

In 1964, Chen et al. obtained samples from patients with KD and showed a myocardial pallor due to patchy necrosis and fibrosis, and sarcolemmal outlines indicative of myocytolysis, a morphology similar to that of white muscle disease (34), a non-inflammatory degenerative muscle disease observed mostly in cattle fed cereals and forage from Se-deficient areas (35). Therefore, some Chinese scientists have considered that high KD incidence may also be associated with Se deficiency. Se as an indispensable trace element that plays an important role in many aspects of human health, such as antioxidant defenses, thyroid hormone metabolism, and the immune system (36, 37). The detection of topsoil Se has suggested that Se concentrations are typically below 0.125 mg/kg, with concentrations of >3 mg/kg in non-endemic areas. Nutritionists have found that the mean Se contents in hair were <0.122 mg/kg in endemic areas and >0.200 mg/kg in non-endemic KD zones (38). In 2020, a study investigated serum Se levels in 571 individuals and found that levels in those living in KD-endemic areas were only 0.97 μmol/L, which was significantly lower than the levels in those living in non-endemic areas (1.01 μmol/L) (39, 40). Further, results of urinary Se loading tests showed that the population in the affected areas was Se-poor (38). The Se status of heart, liver, kidney, and muscle compared with that of individuals with KD was up to 10-fold lower (40, 41). Importantly, support for the Se deficiency hypothesis came from the observation that long-term oral supplementation of an inorganic Se compound, selenite, was effective in reversing the disease in endemic areas (42).

**Figure 4:**
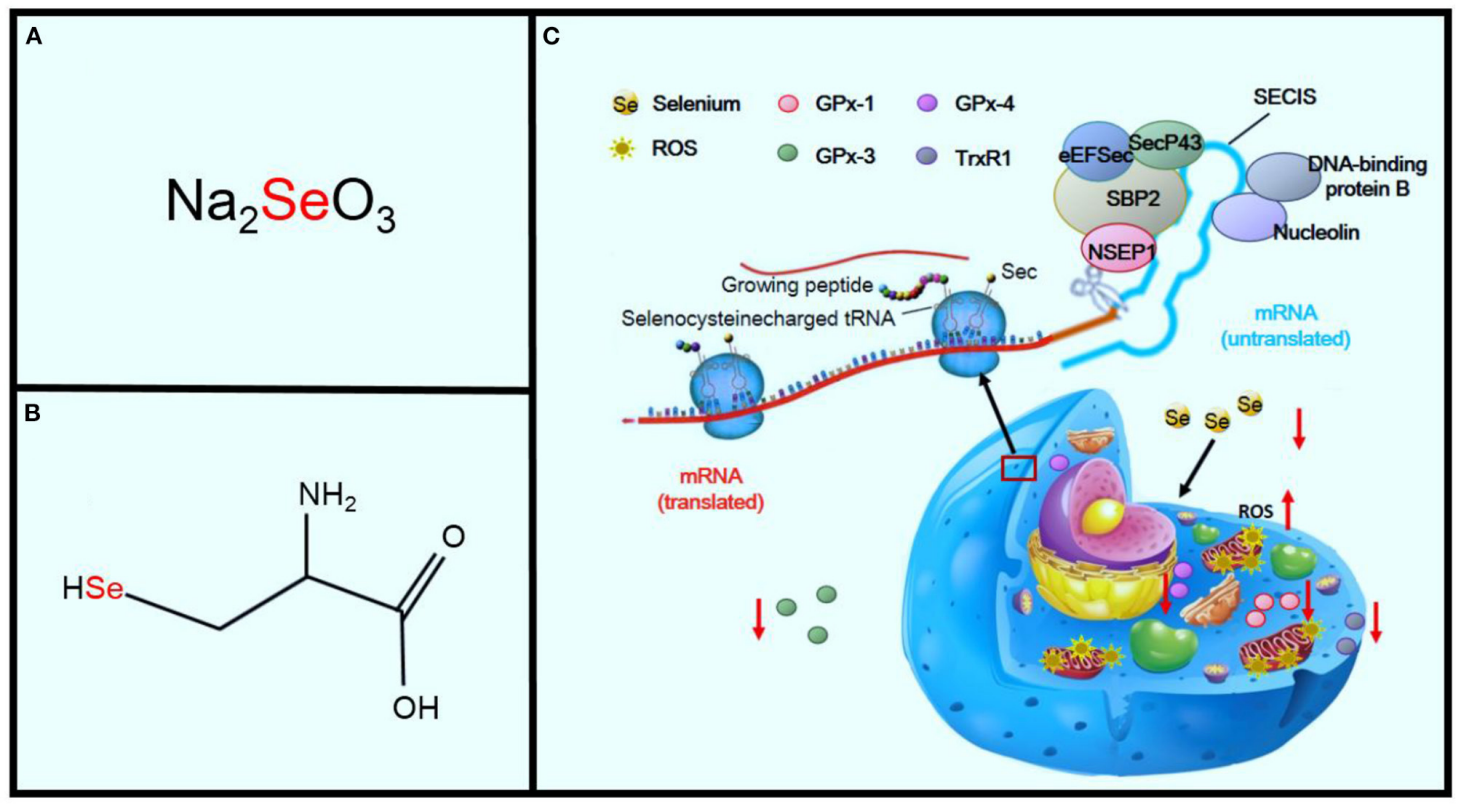
Effects of Se deficiency on KD. Panel **(A)** shows sodium selenite, the primary inorganic form of SE. Panel **(B)** shows the amino acid, l-selenocysteine. Panel **(C)** shows the sequence of mechanisms leading from Se deficiency to oxidative stress in the cell.

Se is an important component of selenoproteins, such as selenoprotein P, deiodinase, and GPx enzymes (43). In 1941, Horn and Jones first showed that Se is incorporated in wheat as an amino acid complex containing sulfur. Subsequently, Trelease and colleagues showed this complex to be Se-methylselenocysteine (Figures 4A,B) (9, 44). Translation of selenoprotein is determined by factors that include the availability of organified Se, a group of translational cofactors, and selenocysteine insertion sequence in the 3′ UTR region of the selenoprotein mRNA (45). Six cofactors include the Sec-specific elongation factor (eEFSec), the selenocysteine-insertion sequence, SECI-binding protein 2 (SBP2), SecP43, nuclease-sensitive element-binding protein 1 (NSEP1), and DNA-binding protein B. Se deficiency seriously affects the synthesis of selenoprotein, and Se is a critical component of a central antioxidant enzyme (41, 45).

GPx and thioredoxin reductase, two selenoproteins, are important members of the body's antioxidant system (46). GPx-1 activity decreases dramatically in Se deficiency and increases during Se supplementation. GPx-1 activity is also associated with GPx-1 (Pro198Leu) polymorphism (47, 48). GPx-1 is the most prevalent of the GPx family and is found in the cytosol of all cells. The deficiency of GPx-1 always exacerbates ischemia-reperfusion injury in cardiomyocytes (49, 50). GPx-3 is a key antioxidant enzyme in the extracellular environment. A deficiency in GPx-3 leads to increased thrombosis as a result of the increased oxidative inactivation of nitric oxide (NO). NO also plays a key role in the regulation of cardiovascular homeostasis and is related to many cardiovascular diseases, including hypertension, atherosclerosis, stroke, and heart failure (51). There is evidence that reactive oxygen species (ROS) are responsible for the reduced NO bioavailability in cardiac and vascular pathologies (52). GPx-4 is an essential regulator of membrane oxidation. A deficiency in GPx-4 decreases proatherothrombotic actions of these peroxidated species. Thioredoxin reductase 1 (TrxR1) is an isozyme of thioredoxin reductase and is an antioxidant enzyme expressed in the cytoplasm (53). It has catalytic reduction activity and thus plays an important role in the oxidative stress reaction. Research has shown that TrxR1 knockout mice showed increased oxidative stress leading to the emergence of heart diseases. Meanwhile, the expression of TrxR1 was significantly lower in cases of KD (54, 55). Se deficiency significantly promotes oxidant stress and injury, which may also potentiate the oxidant injury of other contributing pathogenic factors, including viral and other infections (Figure 4C) (56).

SE deficiency can also lead to other cardiomyopathies, such as dilated cardiomyopathy (DCM) (57). In 2016, a Galveston (TX) investigator reported a rare case of DCM caused by severe malnutrition combined with Se deficiency in a 14-year-old boy (58). The echocardiogram (echo) showed a globular dilated left ventricle with a severely depressed systolic function [ejection fraction (EF) <25%] in accordance with DCM. The boy was initially treated with furosemide and enalapril, and carvedilol was added a week later, since there was minimal improvement in cardiac function, in addition to Se replacement [200 μg (2.5 μmol) twice daily intravenously]. His cardiac function dramatically improved [EF 46%] within 1 week. Two weeks later, his Se level significantly improved. The cardiac function normalized within 4 weeks (59).

## Viral Infections

Enteroviruses are the most common pathogens in human viral myocarditis and are also associated with some DCMs (60). CVB3 is included in the genus *Enterovirus* within the family *Picornaviridae* and is a major causative agent of cardiac muscle infection, but the mechanism is still unclear (61). More recent studies have suggested that CVB3 may be a contributing factor in KD and that Se deficiency has additional, wide-ranging effects (62). Li et al. detected the CVB3 RNA by *in situ* hybridization (3). They found that the positive rates in patients with acute, subacute, and chronic types of KD were 83, 67, and 80%, respectively (63). Their results confirmed that CVB3 might play a key role in the pathogenesis of KD (64). In addition, the distribution of the positive signal of CVB3 was related to the occurrence of KD. CVB3 in acute and subacute KD has been located in the surviving myocardium in or around the necrotic focus, but is dispersed in all cardiomyocytes in chronic KD (65, 66).

**Figure 5:**
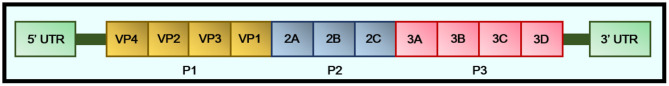
Gene structure of coxsackievirus B3.

The genome of CVB3 is a single-stranded, polyadenylated RNA molecule consisting of 7,400 nucleotides (Figure 5). The single open reading frame (ORF) is located on the side of 5′ UTR and 3′ UTR and is divided into three areas, P1 to P3 (67). The P1 region encodes viral capsid proteins (VP1, VP2, VP3, and VP4) (68). The P2 and P3 regions encode non-structural viral proteins that are important for processing, replication, and translation of multiple proteins. Some studies have shown that the 5′ UTR enteroviruses may play a key role in generating or maintaining myocardial virulence, since the U → C mutation of nt 234 in CVB3 5′ UTR will lead to the attenuated cardiac toxicity phenotype in mice (69). Subsequent analysis of various clinical CVB3 isolates and other enteroviruses indicated that nt 234 is always U regardless of the cardiovirulence phenotype of the virus, consistent with 234C being an artificial mutation (70). This result indicates that the mutation of the virus genome might play an important role in the pathogenesis of KD.

Although the enterovirus infection rate is remarkably high in the myocardial tissue of patients with KD, the hypothesis of viral infection is still questioned (63, 65), because the enterovirus infection phenomenon can also be found in patients without myocardial damage. Recently, research has reported that, in some cases, such as trace element deficiency, a non-cardiovirulent strain can change into a cardiovirulent strain or, under these conditions, can increase cardiac toxicity. The accelerated mutations seen in CVB in Se-deficient mice are the result of the interaction of three factors: rapid replication, lack of proofreading capability, and increased oxidative damage to RNA (17). Viral infections that combine environmental Se deficiency with dietary vitamin E deficiency may explain both the endemic and seasonal characteristics of KD (71).

## Mycotoxin Etiology

The etiology of KD is extremely complex, involving multiple accelerating factors, as well as direct or indirect stimulating factors (72). Recently, Sun et al. suggested that mycotoxins such as *Citreoviridin* and *Fusarium* may initiate KD mainly through oxidative stress mechanisms by the long-term consumption of moldy cereals (72). Dietary deficiency of Se, proteins, and trace elements may act to enhance pathological damage (73).

**Figure 6:**
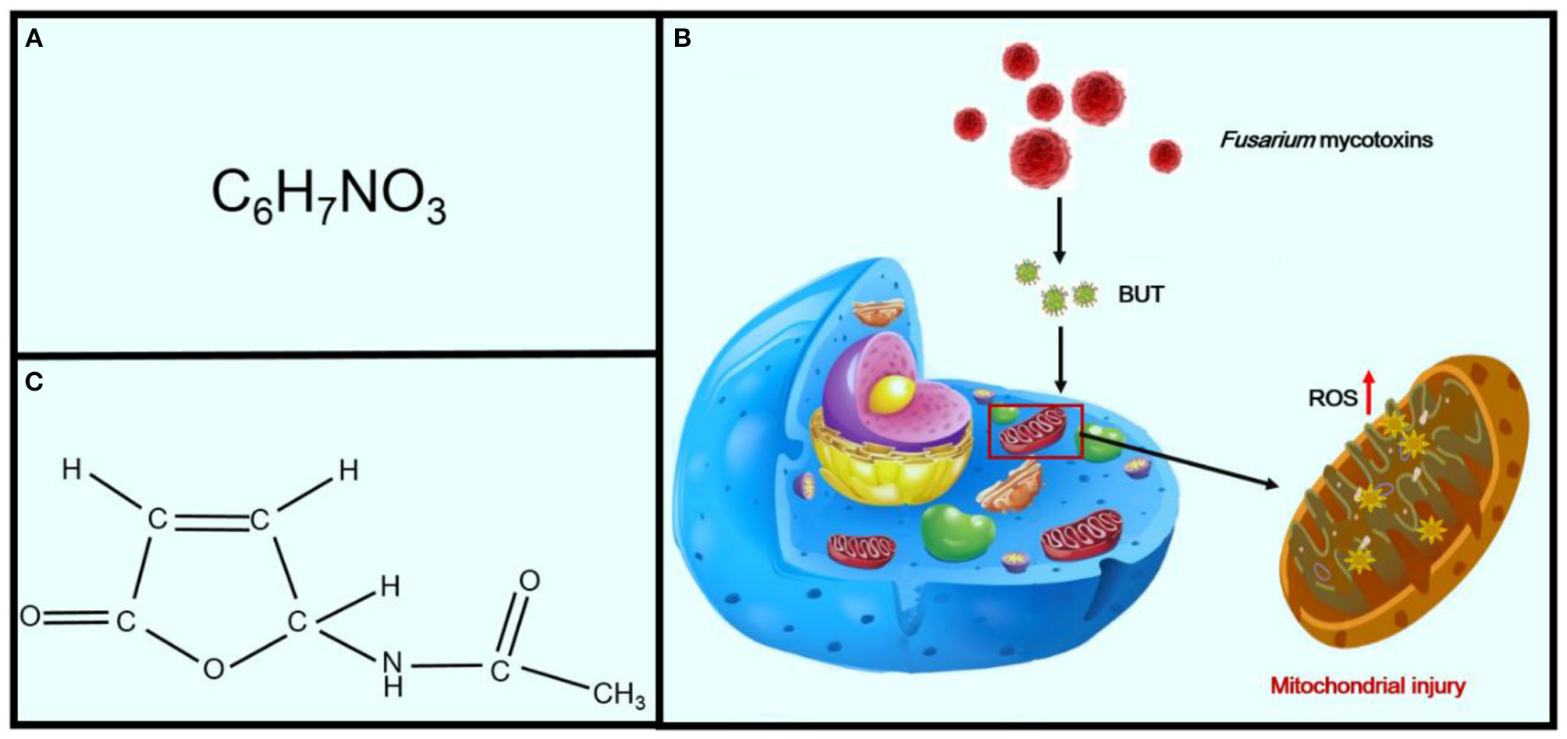
Effects of *Fusarium* mycotoxins on KD. Panel **(A)** shows the chemical formula of BUT. Panel **(B)** shows the chemical structural formula of BUT. Panel **(C)** shows the sequence of mechanisms leading from *Fusarium* mycotoxins to mitochondrial injury in cells.

*Fusarium* mycotoxins, the secondary metabolites of toxigenic *Fusarium* species, are ubiquitously distributed throughout the world. These fungi can produce a variety of *Fusarium* mycotoxins, such as *trichothecenes, fumonisins*, and *zearalenone*, and are frequently detected in foodstuffs and cereals (74, 75). Butenolide (4-acetamido-4-hydroxy-2-butenoic acid γ-lactone; BUT) is one of the most common of the *Fusarium* mycotoxins, which are found in cereals from KD-endemic areas (Figures 6A,B) (75). It was first isolated in 1971 by Yates from *Fusarium tricinctum* NRRL 3249 (76). Increasing evidence has indicated that treatment of rats with BUT (10 and 20 mg/kg per day) for 2 months induces serious myocardial injuries, which are characterized by fragmentation of myofibers and necrosis of the myocardium (74). BUT can also induce a variety of cytotoxicities, such as swelling of the mitochondria, fragmentation of cristae, and phospholipid bilayer rupture, all of which are similar to the characteristics of mitochondrial injuries in patients with KD, leading to speculation that BUT may be one of the etiological factors for KD and that oxidative damage may be essential for myocardial mitochondria (Figure 6C) (77). The mitochondrial respiratory chain is the main source of ROS (78). Furthermore, excessive generation of ROS will attack unsaturated fatty acids in mitochondrial membrane lipids and exacerbate mitochondrial damage (79). Therefore, the mitochondrial lipid peroxidation observed in this study may be the consequence of ROS attack produced by mitochondria, eventually leading to damage to cardiomyocytes (80, 81).

## Clinical Manifestations

Patients with KD are categorized into four types: acute, subacute, chronic, and latent (Table 1).

**Table 1:** Clinical manifestations of four types KD.

**Type**	**Symptoms**	**ECG**
Acute KD	Dizziness, malaise, nausea, loss of appetite, chilly sensation, projectile vomiting, precardia, or substernal discomfort and dyspnea Cardiogenic shock, pallor, constricted veins in the extremities, and low arterial pressure (<80/60 mm Hg)	Proximal tachycardia diminished voltage of QRS waves, prolongation in the atrioventricular conduction time and Q–T intervals, A–V Block, right bundle-branch block, changes in S–T segment and inversion of T wave
Chronic KD	Palpitation, shortness of breath, cough with hemoptysis, pain in the right upper quadrant, edema, and oliguria Enlargement of the heart, reduced intensity of heart sound, a relatively soft and changeable systolic murmur, gallop rhythm, rales on the base of the lungs, hepatomegaly, and edema	Proximal tachycardia (ventricular or supraventricular), arterial fibrillation, frequent ventricular premature beats, bundle-branch block, A–V block, changes in ST–T segment and T wave, enlarged heart forming a flask shape with weak pulsation
Subacute KD	Malaise, restlessness, gallop rhythm, facial edema Slight dilation of the heart, cardiac shock	Bundle-branch block, A–V block, changes in ST–T segment and T wave
Latent KD	Dizziness, fatigue, palpitation Mildly enlarged heart	Right bundle-branch block, first- or second-degree A–V block, and infrequent premature ventricular contractions

### Acute KD

The onset is rapid. Patients present acute heart failure, severe arrhythmia, cardiogenic shock, cardiogenic fainting, and acute pulmonary symptoms and may have cardiomegaly (82). Fibrosis is rare. The most common clinical manifestations include dizziness, loss of appetite, malaise, nausea, chilly sensations, substernal discomfort, and dyspnea. The main physiological symptoms of this type of KD are those caused by cardiogenic shock, such as pallor, venous stenosis of the extremities, and low arterial pressure (<80/60 mm Hg). After control of electric shock, the characteristics of congestive heart failure are always obvious (83). When the heartbeat is <40 beats per min, Adams-Stokes syndrome, caused by atrioventricular block, is not uncommon. In addition, ECG also shows numerous significant changes, such as reduced QRS wave voltage, prolongation in atrioventricular conduction time and Q–T intervals, proximal tachycardia, right bundle-branch block, changes in S–T segments, and inversion of T waves (84). Mild to moderate enlargement of the heart with weakened heartbeat is always shown by roentgenography (85).

### Subacute KD

The onset is slower than that of acute KD, and patients may suffer congestive heart failure or cardiogenic shock shortly after onset. Subacute KD often occurs in children from 2 to 5 years of age (86). The symptoms may be similar to those of common cardiac hypertrophy and marked heart expansion, but myocardial necrosis is not as severe and common as that of acute KD. The onset time of subacute KD is mainly from March to May in the north and from May to August in the south (87). Only a few cases of acute or subacute KD onset have been seen in recent years, mainly in chronic and latent-type patients found in KD-endemic areas (1).

### Chronic KD

This is usually characterized by insidious onset and slow progression. Chronic cases may appear spontaneously or as the consequence of acute and subacute types (88). The symptoms of chronic KD usually vary according to the degree of cardiac insufficiency. The patients present chronic heart failure, congestive heart failure, dilated chambers of the heart, and severe myocardial fibrosis. The main manifestations of the patients include unconsciousness, cough with hemoptysis, shortness of breath, oliguria, and edema. The changes in ECG are even more pronounced in chronic cases, such as atrial fibrillation and right bundle-branch block with left anterior hemiblock (89).

### Latent KD

The onset is disguised, and these types of patients usually have good heart function (NYHA class I). The patient may not even be aware of the disease until death, when it may be discovered only as an incidental finding after a regular physical examination or routine autopsy. The most common complaints are dizziness, fatigue, and heart palpitations after physical activity or work. These symptoms are associated with a minor enlarged heart and abnormal ECG changes, including repolarization abnormalities, such as wide QRS/T angle, QT prolongation, and high QRS non-dipolar voltage. Ventricular extrasystole and right bundle-branch block or ST–T changes are common (90). Cardiomegaly is rarely observed in latent KD, but some minor cardiac abnormalities, with compensating heart function, are always involved.

## Treatment of KD

### Acute KD

For acute cases, mortality can be significantly reduced by early treatment, although there is no specific therapy. Xian Medical College first introduced a regimen whereby large doses of ascorbic acid administered intravenously were found to be an effective treatment for cardiogenic shock. The first dose of 5–10 g, with not <30 g within 24 h, is administered to adults by intravenous injection. Children under 10 years old are given up to half this dose (40). The dosage is repeated for 2 or 3 days and then is decreased slowly as symptoms abate. Moreover, Coenzyme Q10 supplementation at 300 mg/d significantly enhances antioxidant enzyme activities and myocardial metabolism (91). To prevent conversion from the acute to the chronic type, patients should take digitalis and ascorbic acid for 15 consecutive days, after arrhythmia and cardiac insufficiency are restored completely (92).

### Chronic and Subacute KD

The main symptoms of these two types of KD are chronic heart failure, arrhythmias, and ventricular or atrial fibrillation (93). Diuretics increase urine output by inhibiting the reabsorption of sodium or chloride ions from renal tubules (94). Therapy with diuretics is currently considered to be the first-line treatment for patients with chronic heart failure and fluid retention (95). Oral digoxin, 0.125–0.25 mg/d, will increase the contractile force of the heart (96). Common diuretics and their doses are shown in the Table 2.

**Table 2:** Common diuretics and their dosage.

**Name**	**Initial dose**	**Common dose**	**Maximum dose**
Furosemide	20–40 mg/d	20–40 mg	120–160 mg
Bumetanide	0.5–1.0 mg/d	1–4 mg	6–8 mg
Torasemide	10 mg/d	10–40 mg	100 mg
Hydrochlorothiazide	12.5–25 mg, qd	25–50 mg	100 mg
Amiloride	2.5 mg, qd–bid	5–10 mg	20 mg
Triamterene	25 mg, qd	100 mg	200 mg
Spironolactone	20 mg, bid	50–100 mg	100 mg
Tolvaptan	7.5–15.0 mg, qd	7.5–30 mg	60 mg

The β-adrenoceptor blockade is a cornerstone for the treatment of congestive heart failure but inhibited sympathetic nervous system activation. It is suitable for NYHA heart function class II–III or NYHA heart function class IV and for relatively stable patients. Common β-adrenoceptor blockades and their doses are shown in the Table 3.

**Table 3:** Common β-blocker and their doses.

**Name**	**Initial dose**	**Target doses**
Metroprolol succinate	23.75 mg, qd	190 mg, qd
Bisoprolol	1.25 mg, qd	10 mg, qd
Carvedilol	6.25 mg, bid	25 mg, bid
Metoprolol tartrate	12.5 mg, bid	100 mg, bid

KD is a clinical syndrome characterized by excessive activation of neuroendocrine hormones and ventricular remodeling (94). Vasodilators can be treated in patients with poor therapeutic effects from diuretics and digitalis, especially in cases of refractory heart failure. Renin–angiotensin–aldosterone system inhibitors contain angiotensin receptor blockers (ARBs), angiotensin-converting enzyme inhibitors (ACEIs), and mineral-corticoid receptor antagonists, vital functions that have been shown to reduce mortality in patients with heart failure and reduced left ventricular EF (97, 98). However, use of these agents may result in high levels of serum potassium and high risk for serious hyperkalemia (99). Intake of dietary potassium should be controlled, and dietary supplements or herbal remedies that may increase hyperkalemia risk should be avoided. Common ACEI and ARB blockades and their doses are shown in the Table 4.

**Table 4:** Common ACEI/ARB and their doses.

**Name**	**Initial dose**	**Target doses**
Captopril	6.25 mg, tid	50 mg, tid
Enalapril	2.5 mg, bid	10 mg, bid
Fosinopril	5 mg, qd	20 mg, qd
Lisinopril	5 mg, qd	10 mg, qd
Perindopril	2 mg, qd	4–8 mg, qd
Ramipril	2.5 mg, qd	10 mg, qd
Benazepril	2.5 mg, qd	10–20 mg, qd
Imidapril	2.5 mg, qd	5–10 mg, qd
Valsartan	40 mg, qd	160 mg, qd
Candesartan	4 mg, qd	16 mg, qd

### Latent KD

Patients with latent KD should monitor their lifestyle, prevent infection, and balance nutrition. At the same time, it is important for such patients to consult their physicians regularly, so that underlying symptoms can be detected and therapy can be initiated as early as possible (21). Additionally, low doses of drugs and Se could also be used to improve myocardial compensation.

## Prevention of KD

As the causes of KD remain unclear, its elimination or prevention depends on economic developments, attention to KD, and the improvement of living standards in endemic areas, all of which are difficult to achieve in a short period of time (6, 96). One successful program for KD prevention is to supply Se salts to the population in KD-endemic areas. Vitamin E has strong antioxidant properties and is involved in the protection of the membrane structure by preventing the free radical attack of unsaturated fatty acids. Nutritional balance should be actively emphasized for those living in KD-endemic areas.

As described previously, KD is most common in rural, remote, mountainous areas in China, where the majority of the population are low-income, poorly educated, and in poor physical health. Most patients with KD are low-income peasants and underserved in the utilization of medical facilities. Therefore, inexpensive and effective treatment is very important for them. The endemic areas' health politics should focus on contributing to disease control and the interruption of disease transmission by (i) relief of treatment costs for KD patients, (ii) improving clinical diagnosis and case management and sharing information about KD, and (iii) training health personnel to facilitate diagnosis and medical care.

## Future Perspectives

Although the etiology of KD is not yet fully clear, Se deficiency is the most convincing hypothesis (41), based primarily on the following: first, low soil Se concentrations in KD-endemic areas lead to deficient nutritional status of Se in local residents through the food chain; second, Se concentrations in urine, blood, and hair of patients with KD and living in endemic areas were significantly lower than those of healthy people living in non-endemic areas (100); third, the Se contents of patients with KD were positively related to the prevalence of KD; and last, the incidence of KD could be decreased by Se supplementation in KD-endemic areas (101).

Similar to KD, Kashin-Beck disease (KBD) always occurs in areas with a low Se eco-environment (102). This disease further confirms that Se deficiency seriously affects human health (103). KBD is an endemic chronic osteochondral disease, with main symptoms including symmetrical enlargement of the phalanges, joint deformity, and even dwarfism (104). SE supplementation measures have been widely implemented in KD- and KBD-endemic areas throughout the country, which has led to a significant decrease in the incidence of KD and KBD (42). Despite the fact that Se deficiency was closely related to the prevalence of both KD and KBD, oversupplementation can have toxic effects on the human body. Characteristic features of Se toxicity include brittle hair, hair loss, and stratified nails, along with an odor of garlic on the breath and skin (19). More acute Se poisoning cases can include pulmonary challenges and vomiting (105). For the effective prevention of KD and Se poisoning, supplementary Se intake should be monitored appropriately.

Is Se deficiency really the etiology of KD? The hypothesis that Se deficiency causes KD is still disputed by many scholars (7). The epidemiological and clinical features of KD are not fully addressed by Se deficiency alone (21). First, not all Se-deficient individuals in the endemic area have the disease. Moreover, cases of KD have been found in some areas with normal Se content, such as Wenshang County in Shandong Province. Second, the incidence of KD cannot be fully controlled by Se supplementation only (20). Further studies investigating the relationship between Se and KD are needed to determine not only Se status but also genotype in relation to selenoproteins and related pathways (106).

Although KD has been known for nearly a century, its therapeutic strategy has been severely hampered by critical shortcomings of donor patient samples and pathological models (107). Therefore, generating human-cell-based functional and personalized disease models on a sufficiently large scale to meet the demand for drug efficacy and toxicity tests is a major challenge that must be overcome. The generation of induced pluripotent stem cells (iPSCs) may provide an efficient platform for pathogenesis research and drug screening. With the advancement of hiPSC technologies, attention has been devoted to the study of an organoid, i.e., a three-dimensional tissue in a dish. Multiple organoids have been successfully developed, such as the myocardium, liver, stomach, and pancreas (108, 109). Recently, a scaffold-free cardiac organoid differentiation from hiPSCs has been reported, which functionally and structurally resembles the lumenized vascular network in the developing myocardium (110). In the future, we intend to reprogram myocardial cells from patients with KD to iPSCs to construct a KD organoid model, which will contribute to the etiology and drug screening of KD.

Since the first case of KD was discovered, early diagnosis has been the focus of KD research. Several medical methods, such as ECG, X-ray, and ultrasonography, are also commonly used for this purpose. However, their specificities are limited, although ultrasonography is useful for distinguishing KD from hypertrophic cardiomyopathy, rheumatic heart disease, and pericarditis. It is difficult to distinguish early stages of KD from idiopathic DCM, as both show an enlarged heart, systolic dysfunction, and arrhythmias (40, 111). Currently, studies have increasingly focused on the use of saliva and serum for screening potential lectins as differential diagnostic biomarkers of patients with KD. Wang et al. demonstrated that *Solanum tuberosum* (potato) lectin (STL) may be used as a biomarker for the diagnosis of male chronic KD and latent KD and female latent KD. *Triticum vulgaris* (WGA) may be useful in distinguishing between the two different stages (28). Subsequently, the content of LDH isoenzyme and HRAb in the serum of patients with KD may be used in the diagnosis of KD. KD is mainly caused by repeated injury to the myocardium. When cardiomyocytes are damaged, LDH isozymes and HRAb may be released into the blood. Thus far, there is no gold standard for the diagnosis of KD, and it is hoped that there will be a major breakthrough in the diagnosis of KD in the near future.

In conclusion, KD remains of great concern in endemic areas in China, and complete eradication of this endemic myocardial disease requires worldwide attention. Thus, an effective approach is essential to address this challenging disease, for better control strategies, the development of new diagnostic tools and medications, and investigation and treatment of different types of KD.

## Author Contributions

FW and YS: study concept and design and drafting of the manuscript. FW, QY, and XC: critical revision and final approval of the manuscript. FW, WY, and XT: administrative and technical support. FW: obtained funding and study supervision. All authors contributed to the article and approved the submitted version.

## Conflict of Interest

The authors declare that the research was conducted in the absence of any commercial or financial relationships that could be construed as a potential conflict of interest.
